# Early Onset of AbobotulinumtoxinA Effects in Post-Stroke Upper Limb Spasticity: A Prospective Observational Pilot Study

**DOI:** 10.3390/toxins18090400

**Published:** 2026-09-18

**Authors:** Riccardo Marvulli, Serena Caforio Montesardo, Chiara Moccia, Marisa Megna, Maurizio Ranieri

**Affiliations:** 1 Department of Translational Biomedicine and Neuroscience (DiBraiN), University of Bari Aldo Moro, G. Cesare Place 11, 70125 Bari, Italy; caforio.serena@gmail.com (S.C.M.); marisa.megna@uniba.it (M.M.); maurizio.ranieri@uniba.it (M.R.); 2 Independent Researcher, 70125 Bari, Italy; chiaramoccia93@gmail.com

**Keywords:** abobotulinumtoxinA, post-stroke spasticity, upper limb spasticity, compound muscle action potential, neurophysiology, rehabilitation

## Abstract

Upper limb spasticity is a common complication of stroke that negatively affects motor function, range of motion, and quality of life. Although abobotulinumtoxinA is widely used for the treatment of focal post-stroke spasticity, evidence regarding its early onset of action remains limited. This prospective observational pilot study investigated the early clinical, functional, and neurophysiological effects of abobotulinumtoxinA in 20 adults with post-stroke upper limb spasticity. Patients received ultrasound-guided injections into the biceps brachii and flexor digitorum superficialis muscles and were evaluated at baseline, 24 h, 5 days, 7 days, 14 days, and 30 days after treatment. Outcome measures included the Modified Ashworth Scale (MAS), passive range of motion (ROM), caregiver-reported hand function assessed using a visual analog scale (VAS), and compound muscle action potential (cMAP) recordings. No significant changes were observed at 24 h. A significant reduction in spasticity was detected at the day-5 assessment, accompanied by significant improvements in ROM and functional outcomes. Neurophysiological assessment demonstrated significant reductions in cMAP amplitudes at the day-5 assessment, indicating early neuromuscular transmission blockade. Clinical improvements were also first detected at day-5 assessment and were maintained at subsequent evaluations through day 30. These findings indicate that clinically meaningful effects of abobotulinumtoxinA were detectable by the first scheduled assessment at day 5 after injection. Given the absence of intermediate assessments between 24 h and day 5, the exact timing of treatment onset cannot be determined. A multimodal assessment approach may help characterize early treatment-related changes and provide useful information for future studies investigating the timing of adjunctive rehabilitation interventions.

## 1. Introduction

Upper limb spasticity is a common complication of stroke, affecting approximately 30–40% of survivors and contributing to pain, reduced range of motion, impaired voluntary movement, functional limitations, and diminished quality of life [1,2]. Spasticity results from lesions involving upper motor neuron pathways and is characterized by a velocity-dependent increase in muscle tone associated with abnormal reflex activity and altered sensorimotor control. If left untreated, it may lead to secondary complications such as muscle shortening, joint contractures, difficulties with personal care, and increased caregiver burden [1,2]. Botulinum toxin type A (BoNT-A) is considered the first-line pharmacological treatment for focal post-stroke spasticity and has demonstrated efficacy in reducing muscle overactivity, improving passive movement, and facilitating rehabilitation interventions [3,4,5]. Among the available formulations, abobotulinumtoxinA (Dysport^®^) is widely used in clinical practice and has demonstrated a favorable efficacy and safety profile across different stages of post-stroke recovery [4,5]. Following intramuscular injection, BoNT-A inhibits acetylcholine release at the neuromuscular junction through the cleavage of synaptosomal-associated protein 25 (SNAP-25), resulting in a reversible reduction in muscle overactivity [6,7,8]. Beyond its peripheral effects, accumulating evidence suggests that BoNT-A may modulate spinal and supraspinal mechanisms involved in spasticity by altering afferent input and sensorimotor integration [9,10,11]. Although the clinical efficacy of BoNT-A is well established, most studies have focused on outcomes assessed several weeks after injection, corresponding to the period of maximal clinical benefit [12,13,14]. Consequently, the temporal profile of the early onset of action of abobotulinumtoxinA remains incompletely characterized. This information is clinically relevant because early identification of treatment response may facilitate individualized rehabilitation planning and optimize the timing of adjunctive therapeutic interventions. Furthermore, conventional clinical scales such as the Modified Ashworth Scale (MAS) may not be sufficiently sensitive to detect subtle early changes in muscle properties [15]. Instrumental and neurophysiological assessments, including compound muscle action potential (cMAP) recordings and quantitative muscle evaluation, may provide objective markers of treatment-related changes before clinically evident improvement becomes apparent [13,16,17,18]. Therefore, the aim of this study was to investigate the early clinical, functional, and neurophysiological effects of abobotulinumtoxinA in patients with post-stroke upper limb spasticity using a multimodal assessment approach. We hypothesized that treatment-related changes could be detected before the achievement of maximal clinical benefit and that clinically meaningful effects would emerge within the first weeks following injection.

## 2. Results

### 2.1. Patient Characteristics

Twenty patients completed all scheduled assessments and were included in the final analysis. No serious treatment-related adverse events were reported during the study period. Baseline demographic and clinical characteristics are summarized in Table 1. Clinical, functional, and neurophysiological outcomes across all study time points are presented in Table 2.

### 2.2. Clinical Spasticity Assessment

**Figure 1:**
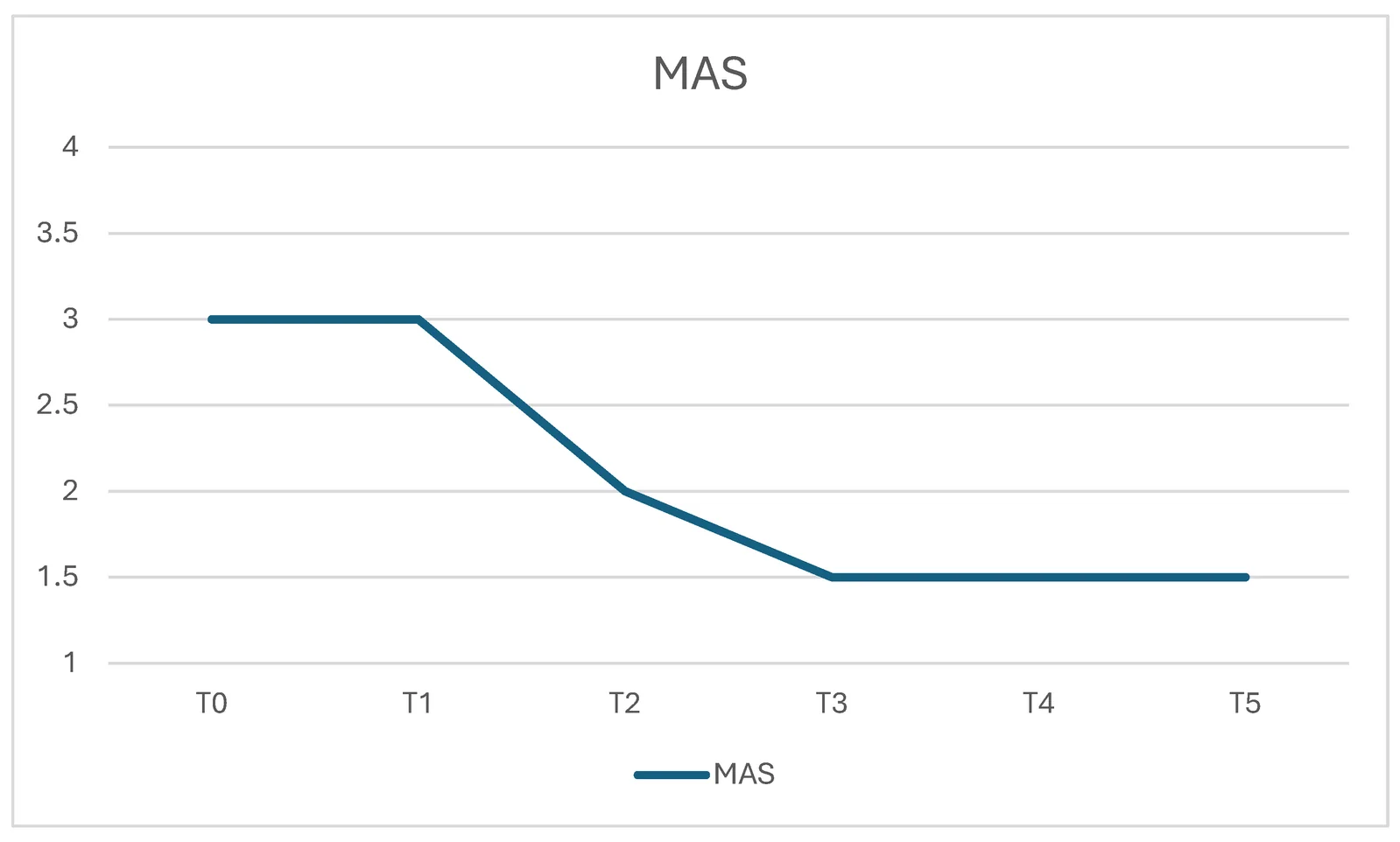
Modified Ashworth Scale (MAS) scores at baseline (T0), 24 h (T1), 5 days (T2), 7 days (T3), 14 days (T4), and 30 days (T5) following abobotulinumtoxinA treatment. Data are presented as median values.

At baseline, the median Modified Ashworth Scale (MAS) score was 3 (range 2–4) for both the biceps brachii and flexor digitorum superficialis. No significant differences were observed 24 h after injection (T1). A significant reduction in spasticity was detected by day 5 (T2), with a median MAS score of 2 (range 2–3) compared with baseline (*p* = 0.0228). Further reductions were observed at day 7 (T3), day 14 (T4), and day 30 (T5), with median scores of 1.5 (range 1.5–3) at all three time points (all *p* = 0.0032 vs. baseline). No significant differences were found between T3, T4, and T5, indicating relatively stable MAS scores across subsequent scheduled assessments (Figure 1).

### 2.3. Passive Range of Motion

**Figure 2:**
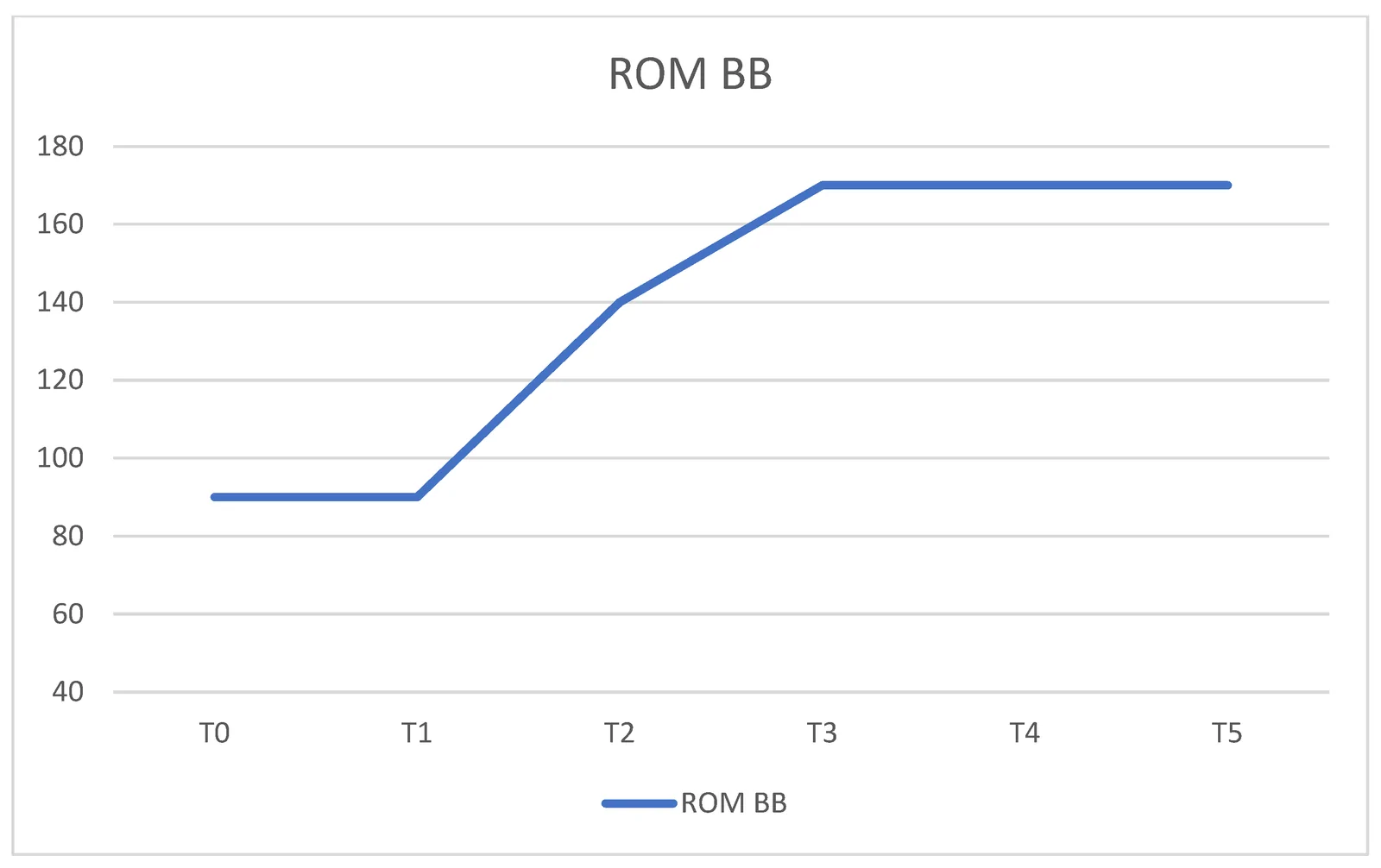
Passive elbow extension range of motion (ROM) at baseline (T0), 24 h (T1), 5 days (T2), 7 days (T3), 14 days (T4), and 30 days (T5) following abobotulinumtoxinA treatment. Data are presented as median values.

Passive elbow extension range of motion (ROM) did not significantly change 24 h after treatment. A significant increase was observed by day 5, reaching a median value of 140° (range 100–180°) compared with 90° (range 70–120°) at baseline (*p* = 0.0012). ROM further improved at day 7 and remained stable at day 14 and day 30, reaching a median value of 170° (range 130–180°) at all subsequent assessments (all *p* = 0.0010 vs. baseline). Pairwise analysis confirmed a significant increase between T1 and T2 (*p* = 0.0012), whereas no significant differences were observed after day 7 (Figure 2).

### 2.4. Functional Outcome Assessment

**Figure 3:**
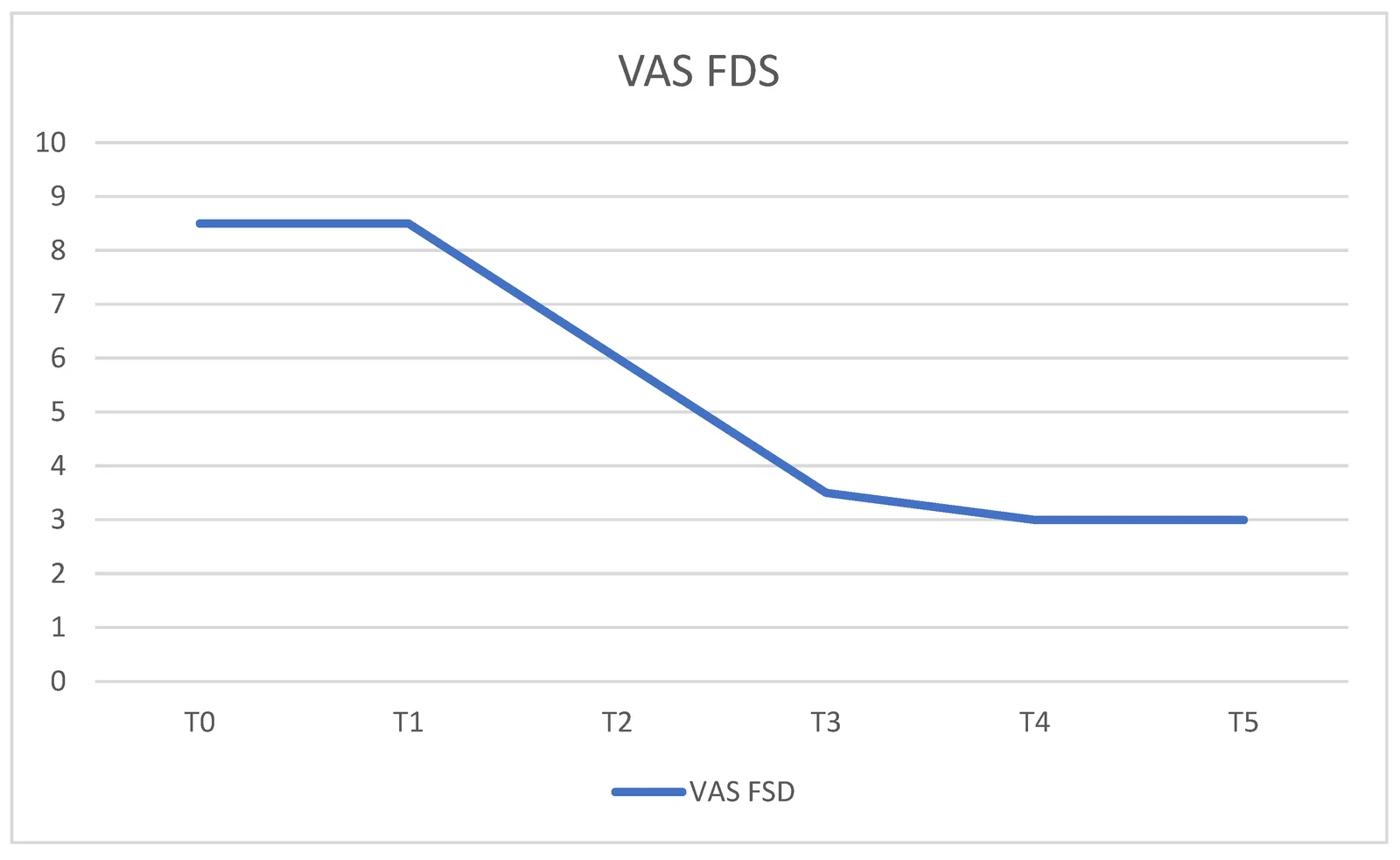
Caregiver-reported hand function scores assessed by visual analog scale (VAS) at baseline (T0), 24 h (T1), 5 days (T2), 7 days (T3), 14 days (T4), and 30 days (T5) following abobotulinumtoxinA treatment. Data are presented as median values.

The median caregiver-reported hand function score assessed by VAS was 8.5 (range 7–10) at baseline. No significant changes were observed at 24 h. Significant improvements were first detected at the day-5 assessment, with median scores decreasing to 6 (range 5–7) at T2 (*p* = 0.0012 vs. baseline). Additional improvements were observed at day 7, with a median score of 3.5 (range 2–6), and remained stable at day 14 and day 30, both showing median values of 3 (range 2–5). Significant differences were observed between T2 and all subsequent assessments, whereas no significant differences were found among T3, T4, and T5 (Figure 3).

### 2.5. Neurophysiological Assessment

No significant changes in cMAP amplitudes were observed 24 h after injection. Significant reductions were first detected at the day-5 assessment, with median cMAP amplitudes decreasing from 13.3 mV (range, 11.0–15.5) at baseline to 11.3 mV (range, 9.0–12.4) at T2 (*p* = 0.0013).

Further reductions were observed at day 7, with a median value of 5.1 mV (range, 4.3–6.3), and remained substantially stable at day 14 and day 30, both showing median values of 4.9 mV (range, 4.2–5.9).

**Figure 4:**
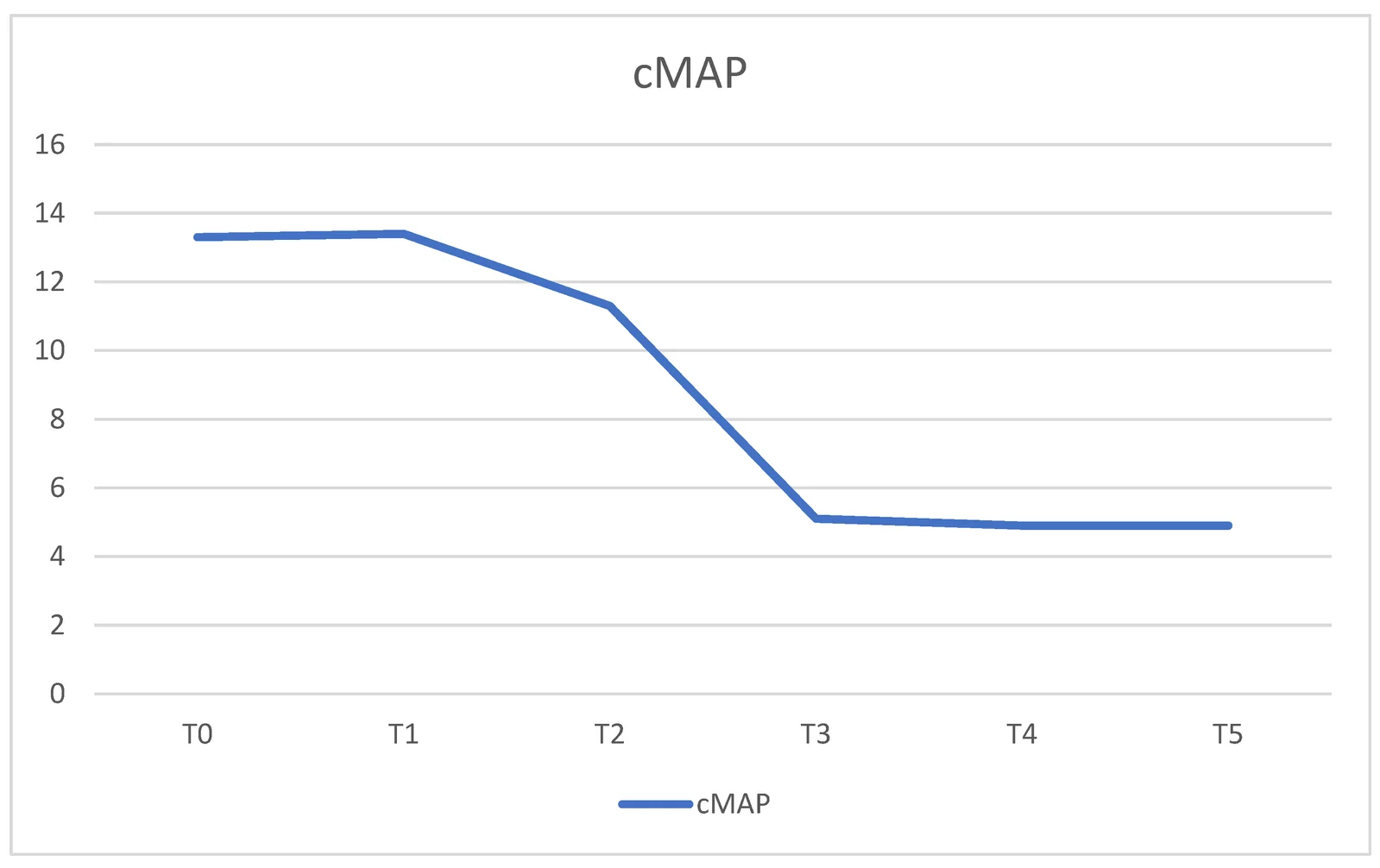
Compound muscle action potential (cMAP) measurements at baseline (T0), 24 h (T1), 5 days (T2), 7 days (T3), 14 days (T4), and 30 days (T5) following abobotulinumtoxinA treatment. Data are presented as median values.

Significant differences were observed between T3 and both T4 and T5 (*p* = 0.0348), whereas no significant difference was found between T4 and T5 (Figure 4).

### 2.6. Temporal Profile of Treatment-Related Changes

Overall, statistically significant clinical, functional, and neurophysiological changes were first detected at the scheduled day-5 assessment. Further changes were observed at day 7, while subsequent assessments showed relatively stable values through day 30. Because no assessments were performed between 24 h and day 5, the exact timing of treatment onset within this interval cannot be determined. Therefore, the present findings characterize the earliest observed treatment-related changes within the predefined assessment schedule rather than establishing the precise onset of action of abobotulinumtoxinA.

## 3. Discussion

The present pilot study investigated early clinical, functional, and neurophysiological changes following abobotulinumtoxinA administration in patients with post-stroke upper limb spasticity using a multimodal assessment approach. The principal finding was that statistically significant changes across clinical, functional, and neurophysiological measures were first detected at the scheduled day-5 assessment and were maintained at subsequent evaluations through day 30. However, because no intermediate assessments were performed between 24 h and day 5, these findings should not be interpreted as establishing day 5 as the precise onset of treatment action. Rather, they indicate that treatment-related changes were already detectable by the day-5 assessment within the predefined evaluation schedule. The temporal profile observed in our cohort is consistent with the established pharmacodynamics of botulinum toxin type A. Following internalization into cholinergic nerve terminals, the toxin cleaves SNAP-25, thereby inhibiting acetylcholine release at the neuromuscular junction and inducing a progressive reduction in muscle overactivity [6,7,19]. This biological process requires several days before clinically detectable effects become apparent, which may explain the absence of significant changes at 24 h and their detection at the subsequent day-5 assessment. Our findings are in agreement with previous studies demonstrating that the onset of BoNT-A activity typically occurs within the first days after injection and precedes the achievement of maximal clinical benefit [12,14,19]. A major strength of the present study lies in the integration of neurophysiological, functional, and clinical outcome measures. Although conventional clinical scales remain the standard approach for the assessment of spasticity, they may be insufficiently sensitive to detect subtle early treatment-related changes [15,20]. In our cohort, cMAP recordings demonstrated significant reductions at the day-5 assessment, paralleling the progressive improvements observed in clinical outcomes. These findings are consistent with previous experimental and clinical investigations showing that cMAP measurements provide objective evidence of BoNT-A-induced neuromuscular transmission blockade [16,17,21]. Although cMAP amplitudes do not directly quantify spasticity, they provide an objective measure of the biological activity of BoNT-A at the neuromuscular junction. Therefore, neurophysiological monitoring may represent a valuable adjunctive tool for the early detection and objective quantification of treatment response. Similarly, significant improvements in passive range of motion and caregiver-reported hand function were first detected at the day-5 assessment and remained stable throughout follow-up. These findings suggest that the reduction in muscle overactivity translated into clinically relevant benefits that may facilitate rehabilitation interventions and improve performance in activities of daily living. The caregiver-reported VAS was selected to capture perceived changes in ease of daily care and hand management during the very early post-treatment phase, when changes detectable by more comprehensive functional scales may be limited. Similarly, despite its recognized limitations, the Modified Ashworth Scale remains the most widely used clinical scale in studies investigating post-stroke spasticity and continues to represent a standard outcome measure in both clinical practice and research. Previous studies have shown that combining botulinum toxin treatment with structured rehabilitation strategies may further enhance functional recovery in patients with post-stroke upper limb spasticity [22,23]. Consequently, these findings may provide a rationale for future studies investigating whether the early identification of treatment-related changes could inform the timing of adjunctive rehabilitation interventions. Several limitations of the present pilot study should be acknowledged. First, the observational design and the absence of a control group preclude definitive attribution of the observed changes exclusively to abobotulinumtoxinA treatment. Second, the relatively small sample size and the absence of an a priori sample-size calculation reflect the exploratory nature of the study and limit the generalizability of the findings. Furthermore, all participants included in the present cohort had experienced an ischemic stroke, which may limit the generalizability of these findings to patients with hemorrhagic stroke. Third, outcome assessors were not blinded to the study time points, which may have introduced assessment bias, particularly for clinical outcome measures. In addition, participants continued concomitant rehabilitation during the study period; therefore, a potential contribution of rehabilitation to the observed clinical and functional changes cannot be excluded. Assessments were focused on two representative upper limb flexor muscles and may not fully capture the complexity of spasticity patterns encountered in routine clinical practice. Finally, the follow-up period was limited to 30 days, precluding evaluation of longer-term clinical and functional outcomes. Future studies should include larger and more heterogeneous patient populations and investigate whether early neurophysiological changes are predictive of long-term functional recovery. Comparative studies involving different botulinum toxin type A formulations may further clarify potential differences in the temporal profile of treatment response and help optimize treatment strategies in clinical practice [24,25].

## 4. Conclusions

AbobotulinumtoxinA demonstrated early clinical activity in post-stroke upper limb spasticity, with significant clinical, functional, and neurophysiological changes first detected at the scheduled day-5 assessment. As no assessments were performed between 24 h and day 5, the precise timing of treatment onset within this interval cannot be established. The observed temporal profile is consistent with the established pharmacodynamics of botulinum toxin type A and was supported by parallel changes in cMAP amplitudes, passive range of motion, and functional outcomes. These findings suggest that a multimodal assessment approach may facilitate the early identification of treatment-related changes and may provide useful information for future studies investigating the timing of adjunctive rehabilitation interventions. Larger controlled studies are warranted to confirm these findings and to investigate whether early neurophysiological changes may have predictive value for longer-term functional recovery.

## 5. Materials and Methods

### 5.1. Study Population

This prospective observational pilot study enrolled 20 adults with post-stroke upper limb spasticity referred to the Neurorehabilitation Unit of Policlinico di Bari. Participants were consecutively recruited among patients referred to the Unit who met the predefined eligibility criteria. Eligible participants were adults (≥18 years) with chronic upper limb spasticity secondary to ischemic stroke, with a time since stroke onset ranging from 6 to 18 months. Exclusion criteria included botulinum toxin treatment within the preceding 6 months, severe cognitive impairment, fixed contractures, ultrasonographic evidence of fibrosis of the target muscles (BB and FDS) and other neurological disorders affecting motor function.

During the study period, participants continued their concomitant rehabilitation program, which included passive and active-assisted mobilization, muscle-strengthening exercises, and motor coordination training.

The study was conducted in accordance with the principles of the Declaration of Helsinki. Ethical approval was obtained from the Institutional Ethics Committee of Policlinico di Bari (approval number: 7948). Written informed consent was obtained from all participants prior to enrollment.

### 5.2. Injection Procedure and Treatment Protocol

All participants received ultrasound-guided injections of abobotulinumtoxinA (Dysport^®^, Ipsen Biopharm Ltd., Wrexham, UK) into the biceps brachii (BB) and flexor digitorum superficialis (FDS) muscles. AbobotulinumtoxinA was supplied in 500-U vials and reconstituted with 2.5 mL of 0.9% saline solution [26,27]. Doses were individualized according to muscle size, severity of spasticity, and individual treatment goals, ranging from 300 to 400 U for the BB and from 250 to 300 U for the FDS.

All injections were performed under real-time ultrasound guidance using a Mindray Z6 ultrasound system (Shenzen Mindray Bio-Medical Electronics CO., Ltd., Shenzen, China) equipped with a linear-array transducer and a 25-G, 1-inch needle. The target muscle was identified sonographically, and needle placement within the muscle was confirmed under direct ultrasound visualization before toxin administration. In the BB, two intramuscular toxin deposits were performed through a single needle entry point, one in the deeper and one in the more superficial portion of the muscle belly, under continuous ultrasound visualization. In the FDS, a single intramuscular toxin deposit was performed under ultrasound guidance.

The BB and FDS were selected as representative proximal and distal target muscles, respectively, because they are commonly involved in the post-stroke upper-limb flexor spasticity pattern. In particular, BB overactivity contributes to resistance to elbow extension, whereas FDS overactivity contributes to resistance to finger extension at the metacarpophalangeal and proximal interphalangeal joints. Ultrasound-guided identification and targeting of these muscles were based on established anatomical principles for proximal and distal upper-limb BoNT-A injections [26,27].

All injection procedures were performed by the same physiatrist with approximately 20 years of experience in botulinum toxin treatment.

Clinical and instrumental assessments were performed at baseline (T0) and at 24 h (T1), 5 days (T2), 7 days (T3), 14 days (T4), and 30 days (T5) following injection.

### 5.3. Outcome Measures

#### 5.3.1. Modified Ashworth Scale

Spasticity was assessed using the Modified Ashworth Scale (MAS), a widely adopted clinical measure of resistance to passive movement. Scores range from 0 to 4, with higher scores indicating greater spasticity.

#### 5.3.2. Passive Range of Motion

Passive elbow extension range of motion (ROM) was measured using a standard goniometer. All measurements were obtained by the same experienced examiner under standardized conditions.

#### 5.3.3. Functional Assessment

Functional outcome was evaluated using a caregiver-reported Visual Analog Scale (VAS) designed to assess the ease of performing hand-related activities of daily living. Scores ranged from 0 (no difficulty) to 10 (maximum difficulty).

#### 5.3.4. Neurophysiological Assessment

Compound muscle action potential (cMAP) amplitudes were recorded from both the biceps brachii (BB) and flexor digitorum superficialis (FDS) muscles using a Galileo NT Line EMG/EP system (EB neuro S.p.A., Florence, Italy; Galileo NT PMS software B8640053000) and Ambu Neuroline concentric needle electrodes (Ambu A/S, Ballerup, Denmark; 25 × 0.30 mm, 30 G). For BB recordings, the musculocutaneous nerve was stimulated, whereas the median nerve was stimulated for FDS recordings. Electrical stimulation was delivered at a frequency of 1 Hz, with stimulus intensity progressively increased until a supramaximal response was obtained, defined as the intensity beyond which no further increase in cMAP amplitude was observed.

At baseline (T0), the stimulation sites were identified to correspond to the injection sites used for each target muscle. The stimulation and recording sites were identified according to anatomical and ultrasound-guided targeting principles, and their positions relative to anatomical landmarks were measured and documented to allow reproducible positioning at subsequent assessments. The same measurements were used at each follow-up visit to reproduce the stimulation and recording sites.

cMAP amplitude was measured peak-to-peak, and cMAP area was simultaneously recorded. All neurophysiological assessments were performed by the same examiner, who was blinded to the clinical outcome results. Skin temperature was not systematically monitored during cMAP recordings. Baseline cMAP measurements on the day of treatment were obtained before abobotulinumtoxinA administration.

Serial cMAP measurements were used as an objective neurophysiological marker of BoNT-A-induced neuromuscular transmission blockade and not as a direct measure of spasticity or functional improvement.

### 5.4. Statistical Analysis

Statistical analyses were performed using R software (version 4.5.1; R Foundation for Statistical Computing, Vienna, Austria). Data distribution was assessed using the Shapiro–Wilk test, with the corresponding W statistics and *p*-values reported in Supplementary Table S1. As the study variables did not consistently meet the assumption of normality across the assessed time points, continuous variables are presented as median (minimum–maximum), whereas categorical variables are presented as counts and percentages.

Changes across study time points were evaluated using Friedman’s test for repeated measures. When significant differences were detected, post hoc pairwise comparisons were performed using the Wilcoxon signed-rank test with Bonferroni correction for multiple comparisons. Statistical significance was defined as *p* < 0.05.

## Figures and Tables

**Table 1:** Baseline demographic characteristics of the study population and abobotulinumtoxinA doses administered to the target muscles (*n* = 20). Data are presented as mean ± standard deviation (SD) or number (%), as appropriate.

Characteristic	Value
Age (years), mean ± SD	65.8 ± 5.98
Male sex, *n* (%)	13 (65)
Female sex, *n* (%)	7 (35)
Stroke type, ischemic, *n* (%)	20 (100%)
Time since stroke onset, months	6–18 (range)
Right-sided spasticity, *n* (%)	6 (30)
Left-sided spasticity, *n* (%)	14 (70)
Biceps brachii dose (U), mean ± SD	349 ± 37.7
Flexor digitorum superficialis dose (U), mean ± SD	279 ± 17.8

**Table 2:** Changes in Modified Ashworth Scale (MAS), passive range of motion (ROM), visual analog scale (VAS) scores, and compound muscle action potential (cMAP) measurements across study time points following abobotulinumtoxinA treatment. Data are presented as median (minimun-maximum). Statistical significance was assessed using Friedman’s test and Wilcoxon signed-rank tests with Bonferroni correction.

	Friedman χ^2^ (df = 5), *p*	Time Point	Median (Range)	Time Point Comparison	Wilcoxon Post Hoc: Bonferroni-Adjusted *p*
**Modified Ashworth Score (0–4)**	78.0, df = 5, *p* < 0.001	t0 (Baseline)	3 (2–4)	t0 vs. t1	-
		t1 (1 day)	3 (2–4)	t0 vs. t2	0.0228
		t2 (5 days)	2 (2–3)	t0 vs. t3, t4, t5	0.0032
		t3 (7 days)	1.5 (1.5–3)	t1 vs. t2	0.0228
		t4 (14 days)	1.5 (1.5–3)	t1 vs. t3, t4, t5	0.0032
		t5 (30 days)	1.5 (1.5–3)		
				t2 vs. t3, t4, t5	0.0118
				t3 vs. t4, t5	-
				t4 vs. t5	-
**Range of Motion**	82.1, df = 5, *p* < 0.001	t0 (Baseline)	90 (70–120)	t0 vs. t1	-
		t1 (1 day)	90 (70–120)	t0 vs. t2	0.0012
		t2 (5 days)	140 (100–180)	t0 vs. t3, t4, t5	0.0010
		t3 (7 days)	170 (130–180)	t1 vs. t2	0.0012
		t4 (14 days)	170 (130–180)	t1 vs. t3, t4, t5	0.0010
		t5 (30 days)	170 (130–180)	t2 vs. t3, t4, t5	0.0741
				t3 vs. t4, t5	-
				t4 vs. t5	-
**Clinical Outcome Affected Limb** **(Score 0–10)**	95.7, df = 5, *p* < 0.001	t0 (Baseline)	8.5 (7–10)	t0 vs. t1	-
		t1 (1 day)	8.5 (7–10)	t0 vs. t2,t3,t4	0.0012
		t2 (5 days)	6 (5–7)	t0 vs. t5	0.0011
		t3 (7 days)	3.5 (2–6)	t1 vs. t2,t3,t4	0.0012
		t4 (14 days)	3 (2–5)	t1 vs. t5	0.0011
		t5 (30 days)	3 (2–5)	t2 vs. t3	0.0017
				t2 vs. t4	0.0012
				t2 vs. t5	0.0011
				t3 vs. t4	0.2691
				t3 vs. t5	0.0503
				t4 vs. t5	1.00
**cMAP**	96.9, df = 5, *p* < 0.001	t0 (Baseline)	13.3 (11.0–15.5)	t0 vs. t1	1.00
		t1 (1 day)	13.4 (11.1–15.5)	t0 vs. t2,t3,t4,t5	0.0013
		t2 (5 days)	11.3 (9–12.4)	t1 vs. t2,t3,t4,t5	0.0013
		t3 (7 days)	5.1 (4.3–6.3)	t2 vs. t3,t4,t5	0.0013
		t4 (14 days)	4.9 (4.2–5.9)	t3 vs. t4,t5	0.0348
		t5 (30 days)	4.9 (4.2–5.9)		
				t4 vs. t5	-

## Data Availability

The data are not publicly available due to privacy and ethical restrictions.

## Supplementary Materials

The following supporting information can be downloaded at: https://www.mdpi.com/article/10.3390/toxins18090400/s1, Table S1: Shapiro–Wilk test statistics (W) and corresponding *p*-values for the study outcome variables across assessment time points.

## Abbreviations

The following abbreviations are used in this manuscript:

ADL	Activities of Daily Living
BoNT-A	Botulinum Neurotoxin Type A
FDS	Flexor Digitorum Superficialis
cMAP	Compound Muscle Action Potential
MAS	Modified Ashworth Scale
ROM	Range of Motion
SNAP-25	Synaptosomal-Associated Protein 25
VAS	Visual Analog Scale
