# Supplementary material for: Early Onset of AbobotulinumtoxinA Effects in Post-Stroke Upper Limb Spasticity: A Prospective Observational Pilot Study
Source: Toxins (Basel). 2026 Sep 18;18(9):400. doi: 10.3390/toxins18090400 (PMC13610992; doi:10.3390/toxins18090400)
Supplement: Supplementary file 1 [file toxins-18-00400-s001.zip › toxins-4447127-supplementary.pdf]

Supplementary Materials: Early Onset of AbobotulinumtoxinA Effects in Post-Stroke Upper Limb Spasticity: A Prospective Observational Pilot Study

Riccardo Giuseppe Marvulli, Serena Caforio Montesardo, Chiara Moccia, Marisa Megna and Maurizio Ranieri

Table S1. Shapiro–Wilk test statistics (W) and corresponding p-values for Modified Ashworth Scale (MAS), Range of Motion (ROM), Visual Analog Scale (VAS), and compound Muscle Action Potential (cMAP) assessed at baseline (T0) and at 1, 5, 7, 14, and 30 days (T1–T5). A p-value < 0.05 was considered indicative of a statistically significant deviation from normality.

Variable	Time point	Shapiro–Wilk W	p-value
**MAS**	T0	0.807	0.001
	T1	0.807	0.001
	T2	0.580	<0.001
	T3	0.658	<0.001
	T4	0.658	<0.001
	T5	0.658	<0.001
**ROM**	T0	0.973	0.809
	T1	0.973	0.809
	T2	0.970	0.745
	T3	0.848	0.005
	T4	0.848	0.005
	T5	0.848	0.005
**VAS**	T0	0.880	0.018
	T1	0.880	0.018
	T2	0.812	0.001
	T3	0.891	0.028
	T4	0.851	0.005
	T5	0.843	0.004
**cMAP**	T0	0.932	0.169
	T1	0.924	0.121
	T2	0.917	0.085
	T3	0.955	0.444
	T4	0.902	0.044
	T5	0.902	0.044
